# Organization of the ventricular zone of the cerebellum

**DOI:** 10.3389/fncel.2022.955550

**Published:** 2022-07-25

**Authors:** Gabriela B. Gómez-González, Marymar Becerra-González, Marianne Lizeth Martínez-Mendoza, Cynthia Alejandra Rodríguez-Arzate, Ataúlfo Martínez-Torres

**Affiliations:** Laboratorio de Neurobiología Molecular y Celular, Instituto de Neurobiología, Universidad Nacional Autónoma de México, Juriquilla, Mexico

**Keywords:** astrocytes, Bergmann glia, choroid plexus, ependymal glial cells, fourth ventricle

## Abstract

The roof of the fourth ventricle (4V) is located on the ventral part of the cerebellum, a region with abundant vascularization and cell heterogeneity that includes tanycyte-like cells that define a peculiar glial niche known as ventromedial cord. This cord is composed of a group of biciliated cells that run along the midline, contacting the ventricular lumen and the subventricular zone. Although the complex morphology of the glial cells composing the cord resembles to tanycytes, cells which are known for its proliferative capacity, scarce or non-proliferative activity has been evidenced in this area. The subventricular zone of the cerebellum includes astrocytes, oligodendrocytes, and neurons whose function has not been extensively studied. This review describes to some extent the phenotypic, morphological, and functional characteristics of the cells that integrate the roof of the 4V, primarily from rodent brains.

## Introduction

The presence of neurogenic niches throughout the ventricular system has stimulated intense research aiming to understand the organization and role of cell groups of glial origin that contact the cerebrospinal fluid and gate relevant information toward and from the brain parenchyma. The roof of the fourth ventricle (4V) is formed by three cerebellar lobules (I, II, and X). Diverse studies have shown little or non-proliferative activity in this area; however, it has been highlighted the presence of heterogeneous cell types expressing GABA-Aρ receptors in the ependymal glial cells (Reyes-Haro et al., 2013; Pétriz et al., 2014), these receptors gate a Cl^−^ channel and desensitize very little upon activation and are insensitive to bicuculline and baclofen. About 60% of the ependymal glial cells respond to GABA by generating Cl^−^ currents insensitive to pentobarbital; these findings supported the view that this area should be formed by a diverse population of cell phenotypes. The cellular diversity was highlighted when an inspection of cellular electrophysiological profiles was conducted in the subventricular zone (SVZ) (Reyes-Haro et al., 2013; González-González et al., 2017); those studies revealed the presence, of astrocytes, oligodendrocytes, neurons and other groups of cells at the ventricular and subventricular cerebellar zones, that remain to be fully described and studied.

The cells that form the wall of the fourth ventricles are crucial for neurogenesis and gliogenesis in early development. During embryonic mouse development, the ventricular zone of the cerebellum is a niche of cells that gives rise to all the neurons of the cerebellum. Individual progenitors are multipotent and produce sequentially different cell types, first to the deep cerebellar nuclei, then the Purkinje neurons and finally to the rest of cerebellar neurons. Also, in the ventricular zone a set of multipotent cortical progenitors is found, these cells produce sequentially cortical neurons, however, lose their potential to produce deep nuclei when longitudinal cell dispersion in the cerebellum becomes limited (Altman and Bayer, 1997; Mathis and Nicolas, 2003). In the mouse embryo (embryonic day 12–14), radial glia fascicles appear at the site of midline fusion at the roof of the fourth ventricle, also radial fascicles appear at the site of midline fusion of the metencephalic plate, radial glia fibers become concentrated within the zone that bounds the germinal trigone which is the site of origin of the external granular layer (Edwards et al., 1990). The early origins of radial glial cells of the cerebellum can be traced to cells of the primitive bipolar cells in ventricular germinal zones. This review focuses on the cell diversity that form the roof of the 4V.

## Diversity of glial cells

The anatomical and functional complexity of the periventricular zone of the cerebellum is derived from the presence of different neuronal types, but also from the heterogeneity of glial cells (Buffo and Rossi, 2013; González-González et al., 2017). According to location, morphology and physiology, the following types of glial cells can be identified in the roof of the 4V: (a) astrocytes, (b) Bergmann glia (BG), (c) nerve-glial antigen two cells (NG2-glia), (d) microglia, and (e) ependymal glial cells (EGCs). The microglia originate from the mesoderm, while the rest share the same ectodermal embryonic origin.

### Astrocytes

Astrocytes, also known as astroglia, are the most abundant and diverse population of glial cells in the central nervous system (CNS). This term was popularized by Santiago Ramón y Cajal who also developed the gold sublimate-method which labeled GFAP (glial fibrillary acidic protein), with this technique it was demonstrated that astrocytes originated from radial glia (Ramóny Cajal, 1916; García-Marín et al., 2007). Astrocytes develop in the embryonic stage and in the late postnatal period (Miale and Sidman, 1961). There are two sites of proliferation in the embryonic ventricular neuroepithelium, (1) the region anterior to the rhombic lip, and (2) the region between the isthmus and the superior cerebellar peduncle (Altman and Bayer, 1987). It is believed that cerebellar glia may be generated by progenitors inhabiting the cerebellar parenchyma (Altman and Bayer, 1987); clonal studies in mouse, showed that astrocytes from the vermis arise from gliogenic radial glia that reaches the ventricular zone in early stages, and at postnatal stages, progenitors from the Purkinje cell layer originate BG and velated astrocytes located in the granular layer (Cerrato et al., 2018).

In general, astrocytes possess multiple primary processes that originate from the soma. In addition, this cell type has intermediate filaments that integrate the cytoskeleton, formed by proteins that define this cell type, including the GFAP and vimentin (Verkhratsky and Butt, 2007). Astrocytes have been classified in protoplasmic and fibrous, according to differences in their morphology and location. Protoplasmic astrocytes are found in the gray matter and exhibit multiple finely branched and complex processes (of ~50 μm long, in rodents). Fibrous astrocytes are found in the white matter and exhibit processes that are longer (300 μm long, in rodents) and less complex compared to protoplasmic astrocytes (Verkhratsky and Butt, 2007).

The cerebellar cortex is formed by two main types of astroglia: Bergmann glia, specialized cells located in the Purkinje cell layer (PCL) and velate astrocytes, which are more common in the granular layer (Farmer and Murai, 2017). Velate astrocytes have a small soma and relatively short processes, where they partially surround the glomeruli and slip between granular cells which synapse with mossy fibers, separating the glomeruli into distinct entities. The velate astrocytes also gather around blood vessels in form of a plaque around the basal lamina forming part of the blood-brain barrier (Landis and Reese, 1982). Velate astrocytes form a syncytial network through gap junctions with nine neighboring cells, giving rise to a highly elaborated and functional coordinated structure in the cerebellum (Kiyoshi et al., 2018).

In the SVZ of the mouse cerebellum, a region delimited dorsally by BG end feet and ventrally by the ependymal cells, the astrocytes are abundant and express GABA-A receptors (Reyes-Haro et al., 2013). A study conducted by Pétriz et al. (2014) showed that GFAP^+^ cells of the granular layer of the cerebellum express GABAρ subunits during early postnatal development. Electrophysiological studies revealed that astrocytes express functional GABA-A receptors, whereas evoked currents were inhibited by bicuculline and TPMPA, indicating the presence of GABAρ subunits. In that study, three populations of GABA-A receptors were identified in astrocytes: classic GABA-A receptors, bicuculline insensitive GABAρ, and GABA-A-GABAρ hybrids. The presence of GABAρ subunits independently or in combination, confers complexity to GABAergic signaling in the area. Also, the modulation of GABAρ dynamics can be a novel extra-synaptic transmission mechanism that regulates GABAergic control of GFAP^+^ cells of the SVZ of the cerebellum in early development (Pétriz et al., 2014).

In recent experiments in which carmustine was administered prenatally, a reduction in the morphology and complexity of astrocytic cells of the 4V of the cerebellum was observed (Rodríguez-Arzate et al., 2021). In a transcriptomic analysis using the well-established hEGP-eGFP reporter mouse line (Nolte et al., 2001), differences were found between protoplasmic cortical astrocytes and cerebellar astrocytes (mainly Bergmann glia). It seems that a specific transcriptional program associated to the Zic/Irx families of transcription factors play a central role in cerebellar astrocytes (Welle et al., 2021).

### Bergmann glia

These cells originate from the radial glia and are a landmark of cerebellum. They are found from early stages of development around embryonic day 15 in mouse, playing an essential role during neurogenesis and cell migration since they form a structural scaffold for cellular migration of granular cells during postnatal development as well as in the dendritic growth of PCs (Bellamy, 2006). In the mouse adult brain, the cell bodies of BGs are ~15 μm diameter and is settled in the Purkinje cell layer, the somas surround these neurons in a proportion of 8 to 1. The BG exhibits multiple processes, with numerous lateral protrusions named microdomains, these processes go through the molecular layer forming a conical terminal foot (Rakic, 1972, 2003). The plane of the processes of BG is organized as a palisade, oriented in parallel to the longitudinal axis of the folia and perpendicular to the plane formed by the dendritic trees of the Purkinje cells (Buffo and Rossi, 2013).

The BG surrounds and covers the dendritic trees of Purkinje neurons, wrapping between 2000 and 6000 synapses (Kettenmann and Ransom, 2005). The BG microdomains respond to synaptic activity by changing Ca^2+^ concentration, which eventually propagates from the processes toward different regions of the cell (Bellamy, 2006). Also, the microdomains are highly dynamic, displaying motility patterns similar to neuronal spines, since they have the capacity to form new processes that grow and retract laterally (Lippman et al., 2008, 2010).

The lateral protuberances of BG processes present glutamate receptors of the α-amino-3-hydroxy-5-methyl-4-isoxazolepropionic (AMPA) subtype, composed exclusively by the GluA1/GluA4 subunits. A study conducted by Saab et al. (2012), demonstrated that BG is essential for synaptic integration and cerebellar output signal and a double knock out of AMPA receptors of the BG leads to deficiencies and alterations of fine motor coordination (Saab et al., 2012).

**Figure 1:**
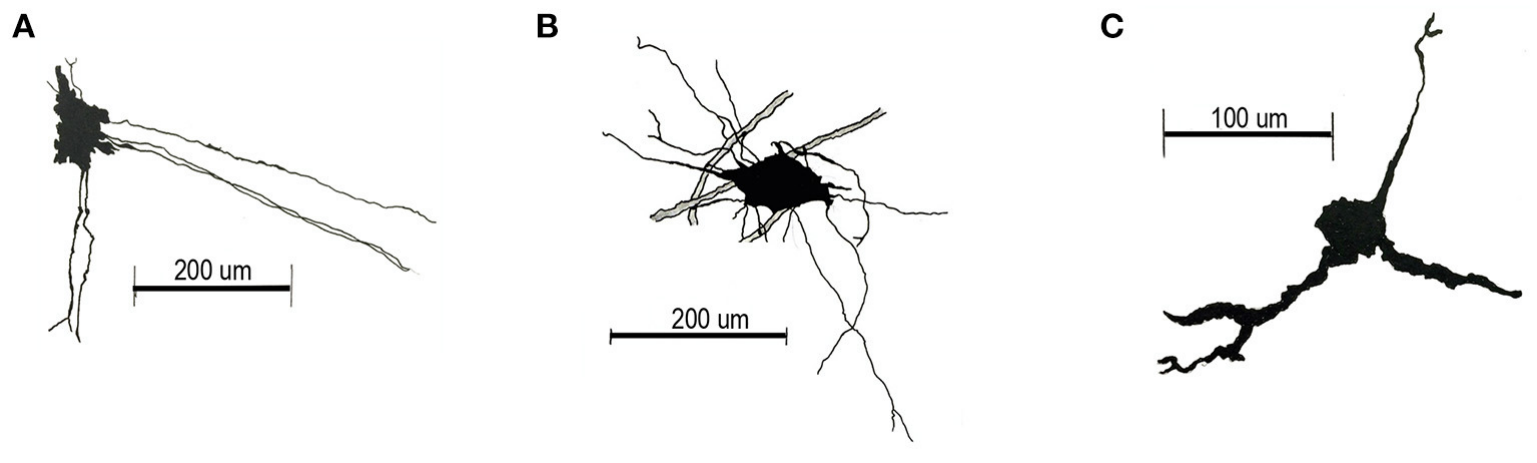
Camera lucida drawings of main cell types of the SVCC. **(A)** Cells located in the lateral region of the SVCC have an elongated soma (diameter 32.506 ± 0.808 μm), long processes that project toward the middle region and processes that extend ventrally. **(B)** Star-shaped soma cells (diameter 36.957 ± 0.774 μm) with a long lateral process and one that projects ventrally. **(C)** Small cells (diameter 17.728 ± 2.223 μm), with two processes projecting ventrally and a short lateral process.

While the BG located in the dorsal cerebellar lobes (lobule III-IX) extend their processes to the pia establishing the *glia limitans* (Yamada et al., 2000), the BG located in the ventral lobules (I, II and X) at the roof of the 4V, projects their processes toward the SVZ, where the end feet limit the upper part of a structure named the subventricular cellular cluster (SVCC) (González-González et al., 2017). The SVCC, extends rostral-caudally along the lobules I and X, and is formed by a diversity of cell types, camera lucida drawing of three sample cells are shown in Figure 1. These cells express GFAP, nestin or both (González-González et al., 2017).

### NG2 glial cells

The nerve-glial antigen two cells (NG2-glia) are also known as oligodendrocyte precursor cells (OPC), NG2 progenitor cells, synantocytes or polydendrocytes (Butt et al., 2002; Nishiyama et al., 2002; Dimou and Gallo, 2015). During the development these cells have different origins (ventral and dorsal ganglionic eminence, SVZ, cerebral cortex), are widespread in the CNS and present diverse morphology (Nishiyama et al., 2002, 2014).

NG2 glial cells are considered the fourth type of glial cells in the central nervous system, these are immature cells with high proliferative capacity and widely distributed throughout the gray and white matter in the postnatal and adult brain, where they make 5–8 of the glial cells population (Dawson et al., 2003; Clarke et al., 2012; Lomoio et al., 2012). NG2-glia can be identified by the expression of several molecular markers, such as platelet-derived growth factor receptor (PDGFRα), Olig2, proteolipid promoter (Plp) (Chung et al., 2013) and Sox 10 (Trotter et al., 2010); besides NG2, which is permanently expressed after birth (Rivers et al., 2008).

In the mouse adult cerebellum, the NG2-glia has a stellate shape and is present in the white and gray matter (Bouslama-Oueghlani et al., 2005; Lomoio et al., 2012; Chung et al., 2013). The general distribution of the NG2-glia in the cerebellum was described in a study conducted by Lomoio et al. (2012) in rats, that showed that NG2 glia at the posterior cerebellar lobules (VI–VIII) is evenly and densely spread in all the cell layers; however, in the anterior and ventral lobes (I–VI and IX–X lobules) most of these cells are in the molecular layer, while in the white matter and the internal granular cell layer (IGL) their presence is sporadic, and decreases with age. NG2-glia from the molecular layer exhibits numerous processes that extend between the dendrites of the Purkinje neurons (Palay and Chan-Palay, 1974; Levine and Card, 1987; Lin et al., 2005), while the NG2-glia from the white matter is highly branched with thin processes and no cell-coupling (Labrada-Moncada et al., 2020).

One of the major roles of the NG2-glia is the generation of myelinating and non-myelinating oligodendrocytes (OL) during postnatal development (P12-P23 in rodents), crucial in the repairment of the myelin loosed under demyelinating pathologies (Watanabe et al., 2002; Nishiyama et al., 2014, 2021), either at the spinal cord or brain (Sherafat et al., 2021). Nevertheless, the fate of NG2-glia as precursor cells has been further investigated *in vivo* using different inducible transgenic mice lines, unveiling that secondary to the OL generation, NG2-glia cells give rise to astrocytes before birth, and this capacity decreases along time (Zhu et al., 2008; Trotter et al., 2010; Nishiyama et al., 2014; Huang et al., 2018). In the adult life, NG2-glia derived astrocytes are found in the gray matter from the forebrain (mainly at ventral cortex, striatum; Zhu et al., 2008) and cerebellum; where the Olig2/Plp positive NG2-glia exhibit a gliogenic fate and generates Bergmann glia cells (postnatal day 7 in mice), however upon hypoxic-ischemic injury the cerebellar oligodendroglial lineage increases while Bergmann cells and astrocytes remain constant (Chung et al., 2013). Evidence strongly suggests the proliferative capacity of NG2-glia cells in response to injury by giving rise to astrocytes, microglia (Sellers et al., 2009) and Schwann cells (Zawadzka et al., 2010). However, despite discussions, most of the evidence support that NG2-glia cells do not generate neurons (Nishiyama et al., 2014; Huang et al., 2018; Sánchez-González et al., 2020).

The multipotential capacity of the NG2-glia is linked to its functional properties that suggest these cells are involved in the proper function of different brain circuits (Akay et al., 2021; Zhang et al., 2021), for instance it has been reported that in the cerebellum these cells express AMPA receptors permeable to Ca^2+^ that sense through synaptic junctions the input from climbing fibers (Lin et al., 2005), and the NG2-glia from white matter expresses functional GABA-A receptors (Labrada-Moncada et al., 2020). On the other hand, cell fate of NG2-glia cells can be modulated by local cues, such as glutamate signaling in the cerebellum (Chung et al., 2013).

### Microglia

Microglia, also known as Hortega cells, who described them in 1932 (Del Rio, 1932), are a population of immune cells that reside in the CNS within the parenchyma associated to the capillary vasculature (Prinz and Mildner, 2011). In the adult murine nervous system, the microglial population is regularly organized as an independent mosaic of cell layers (Lawson et al., 1990). Depending on the region analyzed microglia represents 5–12% of the total number of glial cells (Lawson et al., 1990), showing higher densities in gray matter than in white matter (Mittelbronn et al., 2001). Microglia presents diversity in its morphology, which is mostly arborized with radial orientation; other microglial cells are elongated and mainly oriented to the white matter while others are amoeboid in the circumventricular organs (Lawson et al., 1990).

These cells play a role as the first line of defense in the nervous system (Prinz and Priller, 2014), participating in phagocytosis, acute responses to injury, release of inflammatory modulators and cytokines, and are important for normal brain development and synaptic plasticity (Prinz and Priller, 2014). In the absence of injury or disease, microglia is in a “resting” state constantly “monitoring” its microenvironment (Gómez-Nicola and Perry, 2015), in this state microglia maintains its soma in a fixed position while its multiple mobile processes allow to explore its surroundings while maintaining a mosaic distribution and organization (Dávalos et al., 2005; Nimmerjahn et al., 2005; Wake et al., 2009). Depending on the specific signals of the modulators of microglial activation after injury detection (Hanisch and Kettenmann, 2007; Prinz and Priller, 2014), the microglia transforms into a “reactive” state, in which the motility of their processes allows its migration to the site of injury (Dávalos et al., 2005; Hanisch and Kettenmann, 2007) generating multiple phenotypes of reactive microglia, which present phenotypes similar to the polarization states of M1 and M2 macrophages (Wang et al., 2013; Prinz and Priller, 2014). Reactive microglia have also been shown to release reactive oxygen species, guanosine triphosphate (GTP), ATP, inflammatory cytokines (IL-1β and IL-6), and tumor necrosis factor (TNF-α) (Pascual et al., 2012; Zhan et al., 2014; Prinz and Priller, 2017).

Unlike the microglia located in the brain cortex, the cerebellar microglia display certain unique characteristics. A study carried out by Tay et al. (2017) showed that microglia isolated from the cerebellum of rodents have a higher expression of immune-related genes and are renewed in a shorter time compared to microglia from other regions of the brain. In the cerebellum, microglia are scarce, and their branches are less complex, having only two main processes extending from the soma. Cerebellar microglia show different morphological and dynamic profiles in the molecular layer, Microglia from Purkinje and granular cell layers show soma motility and a dynamic interaction with Purkinje neurons (Stowell et al., 2017). Yamamoto et al. (2019) showed the interaction between microglia and cerebellar neurons through TNF-α signaling, which induced long-term potentiation and thus modulation of motor behavior.

### Ependymal glial cells

The ependyma is a specialized epithelial tissue derived from the radial glia (Spassky et al., 2005), hence these are glial cells with a cuboid shape and multi-ciliated simple columnar form lining the ventricles and interconnected by either gap junction or zonula adherens (Bruni and Anderson, 1987; Oda and Nakanishi, 1987; Telano and Baker, 2021). A network of modified ependymal cells known as the choroid plexus (see following section) produces 70–80% of the cerebrospinal fluid (CSF). EGCs are covered by cilia and microvilli on its apical side, permitting CSF circulation and absorption. Damage to the ependyma may result in low volume of CSF which is also caused by age-related atrophy of ependymal cells (Oda and Nakanishi, 1987; Telano and Baker, 2021; Javed et al., 2022).

Other functions of the EGCs include roles in barrier formation, transport of ions, molecules and water between the CSF and the parenchyma (Albors et al., 2022). The EGCs surround clusters of fenestrated capillaries that permit the filtration of plasma (Telano and Baker, 2021). EGCs are a key component of the subventricular zone stem cell niche, such as in the spinal cord, where some of the EGC lining the central canal are in contact with neural stem cells (Albors et al., 2022). The transcriptomic profile of EGCs is quite diverse in the ventricular system, but all express the gene FoxJ1, which is critical for the production of cilia (Zeisel et al., 2018; Albors et al., 2022), however at the moment there is not a comprehensive transcriptome of ependymal cells of the 4V to help to understand the morphological diversity in this area that includes tanycyte-like cells and biciliated cells in the midline of the cerebellum (González-González et al., 2017).

The surface of the cells that form the wall of the roof of the 4V has been observed under the scanning electron microscope (Oda and Nakanishi, 1987; Alvarez-Morujo et al., 1992). The anterior section is divided in territories rich in cilia, other territories show only a few cilia while others are covered by microvilli but not cilia (Alvarez-Morujo et al., 1992). In addition, numerous spherical bulbs of unknown function face the lumen (Alvarez-Morujo et al., 1992; González-González et al., 2017). Transmission electron microscopy showed that cilia exhibit a 9 + 2 microtubule pattern, and some cilia have a “dilated end”, possibly related with a receptor like function (Alvarez-Morujo et al., 1992; González-González et al., 2017). Along the midline of the ventricle a formation named the ventromedial cord includes biciliated cells intermingled with EGCs. These cells express GFAP, vimentin and nestin and respond to hypoxic conditions by transiently regulating the expression of GFAP (González-González et al., 2017; Becerra-González et al., 2020).

In the EGCs, GFAP expression is related to the state of differentiation during ontogeny and is an indicator of different levels of functional maturity, nevertheless, in adulthood a variation of GFAP expression correlates with diverse functional states. During the postnatal life, EGC's decrease the expression of GFAP but rebounds in the mature brain. Interestingly, EGC's are mitotically active in the adult brain and therefore are in risk of developing tumors (Leonhardt et al., 1987).

In the cerebellum, EGCs express GABA-A receptors with different characteristics from those expressed in neurons, this includes the presence of the GABA-Aρ receptors that confer resistance to bicuculline and insensitivity to pentobarbital (Reyes-Haro et al., 2013). Under the scanning electron microscope ependymal EGCs of the roof of the 4V show diverse features: (1) cells with numerous microvilli and cilia, (2) cells with numerous microvilli and few cilia, located next to the choroid plexus, and (3) cells with sparse microvilli and one or a few cilia (Oda and Nakanishi, 1987; González-González et al., 2017). Within the later, there are some biciliated cells that correspond to a group of EGCs compacted and ordered medially in antero-posterior direction in the roof of the 4V. These cells co-express vimentin and nestin (a marker commonly used to detect precursor cells) that form the ventromedial cord (VMC) which is present since embryonic development and through adulthood (González-González et al., 2017). Some insights about the functionality of these tanycyte-like cells that form the VMC have been revealed by using a model of hypoxic preconditioning in mice, in which expression of GFAP showed a transient reduction upon induction of hypoxic pre-conditioning. In addition, expression of nestin was upregulated in the whole cerebellum but incorporation of BrdU was not detected in the EGCs of the area. As the VMC EGCs responded to this mild stimulation (hypoxic preconditioning), it is suggested that VMC GFAP-positive cells may relay chemical information from the CSF to underlying neural circuits such as the ones extending along the floor of the 4V (Mirzadeh et al., 2017; Becerra-González et al., 2020). It is clear that the diversity of EGCs of the cerebellum and the entire CNS points out to specializations of their functional role, and a map of the distribution of every phenotype will certainly help to understand their selective role according to their distribution along the surface of the 4V.

## Choroid plexus

Four choroid plexuses reside inside the ventricular system of the brain: one in each of the two lateral ventricles, one in the third ventricle, and one in the 4V. Their most important function is the production of the cerebrospinal fluid (CSF). The CSF is formed at a rate of ~4 ml/min per gram of choroid plexus tissue, in the rat, which is ~10 times higher than the rate of the blood supply to the brain parenchyma (Keep and Jones, 1990). The total volume of CSF in the entire human CNS amounts about 150 ml, and ~500–600 ml is produced in 24 h; thus, the CSF is replaced three to four times per day (Cserr, 1971).

The choroid plexus of the 4V is T-shaped, extended toward the lateral angles of the rhomboid shaped roof and its vertical limb extends caudally. It is composed by a single layer of cuboidal epithelial cells, with numerous club-shaped microvilli and occasional short cilia at the ventricular surface. Choroid cells are connected by tight and gap junctions that form a *zonula adherens* (Oda and Nakanishi, 1987).

Epithelial cells are the predominant cell-type in the choroid plexus (Keep et al., 1986; Keep and Jones, 1990), at the caudal end of the 4V the epithelial cells surround 1–4 cell clusters composed of 15–20 cells that form structures named “choroid bodies” whose function is not known yet (Levine and Saltzman, 2003). Transcriptomic studies have revealed the expression of different coding and non-coding regulatory mRNAs in the choroid plexus. A comparative transcriptomic analysis of the choroid plexus from the lateral and fourth ventricles (from mice, macaque, and human) revealed specialized domains of secretory cells that predict that cells from both regions contribute differently to cell-signaling to the CSF (Lun et al., 2015). One interesting signaling molecule produced by the choroid plexus of the 4V is the bone morphogenic protein or BMP, which negatively modulates neuron differentiation of rhombic lip-derived cells, that gives rise to cerebellar granular neurons (Krizhanovsky and Ben-Arie, 2006). A non-coding RNA expressed in the choroid plexus of the 4V is the microRNA (miRNA) 449. miR449 is expressed in the epithelial cells of the choroid plexus in the 4V in developing and adult mouse (Redshaw et al., 2009) and is known to regulate ciliogenesis by inhibiting the Notch pathway. If the molecular circuit downstream of miR449 is perturbed it may lead to ciliopathies (Marcet et al., 2011).

Due to the inaccessibility of the plexus choroid cells within the 4V, experimental analysis of their electrophysiological properties has been very limited. Whole-cell patch-clamp of choroid plexus epithelial cells maintained in culture have shown a lower mean capacitance than those from the 3V (55 vs. 61 pF) and express several conductances that include a delayed-rectifying potassium channel, an inwardly rectifying chloride channel and a volume sensitive anion channel, which are very similar to those of the lateral ventricle (Kibble et al., 1996; Speake and Brown, 2004). Epithelial cells of the choroid plexus present ion-transport proteins that mediate the transcellular movement of molecules from the blood to the CSF and maintenance of ion concentrations is important for a continuous regulation of cell volume of these cells that are immersed in the CSF. Control of cell volume of choroid plexus epithelial cells of the 4V depend on a Na^+^-K^+^ exchanger and a Cl^−^-${\text{HCO}}_{3}^{-}$ exchanger that confer the capacity to respond to variations in extracellular osmolality (Hughes et al., 2010).

The temporal coordination of internal biological processes is crucial to mammals. A complex network of cell independent oscillators is found in different tissues and diverse studies indicate that the suprachiasmatic nucleus of the hypothalamus is the master circadian clock (Cheng and Cheng, 2021; Lu and Kim, 2021). Expression of critical clock genes and their products has been detected in the cells of the choroid plexus, which have a circadian oscillator modulated by estrogens (Quintela et al., 2013, 2018; Yamaguchi et al., 2020) and in explants of choroid plexus maintained *in vitro*, including from the 4V, the circadian rhythmicity remained and even exceeded that of the suprachiasmatic nucleus (Myung et al., 2018). Thus, the choroid plexus seems to affect the master clock in the suprachiasmatic nucleus most probably by biochemical signaling through the cerebrospinal fluid (Quintela et al., 2021).

Novel brain regenerative strategies apply transplantation of cells to induce functional rescue of brain lesions caused by trauma or neurodegenerative diseases. Cell therapy appears to increase neurotrophic factors levels and reduce neuroinflammation and several studies have demonstrated the potential of choroid plexus cells to reduce neurological deficits and promote neuroregeneration. For example, cells from the choroid plexus of the 3V and 4V were grafted in the 4V of rats with ischemic brain injury. This strategy reduced neurological deficits and infraction volume, it also reduced the number of apoptotic and inflammatory cells (Matsumoto et al., 2010). Furthermore, choroid plexus cells from the 3V and 4V promoted axonal extension and tissue repair in an experimental model of spinal cord lesion, in which axonal extension of the injured tissue was observed after transplantation (Kanekiyo et al., 2016), grafted cells also gave rise to astrocytes in lesioned spinal cord mice (Kitada et al., 2001) and induced nerve regeneration in the dorsal funiculus of rat spinal cord by supporting a massive growth of axons (Ide et al., 2001). All these effects of the grafted follicular cells may be associated to the expression and release of different neurotrophic factors that affect the local environment of the recipient tissue, although some neurogenic activity is not fully discarded in choroid plexus of the 4V (Itokazu et al., 2006).

## Vascular organization

The vertebral arteries enter the cranium trough the *foramen magnum* to supply the cerebellum and the brainstem with blood, carrying oxygen, nutrients and hormones obtained from the cerebrospinal fluid (Kulik et al., 2008; Cipolla, 2009; Fogwe and Mesfin, 2018), maintaining homeostasis. Three arteries supply the cerebellum: (1) the *superior cerebellar artery*, branching from the lateral portion of the *basilar artery* from which, (2) the *anterior inferior cerebellar artery* also branches, and (3) the *posterior inferior cerebellar artery* branching from the vertebral arteries, inferior to their junction with the basilar artery.

Vascularization occurs by angiogenesis as in the rest of the CNS, the cerebellar angioarchitecture of mouse starts its differentiation at embryonic day 9.5, from a blood vessel that arrives from the perineural vascular network. Vascular sprouts grow radially into the hindbrain parenchyma and by embryonic day 10.5 they are able to change their growth directionality to fuse with other sprouts and form a highly stereotyped vascular network named the *subventricular vascular plexus* with a honeycomb shape (Cipolla, 2009; Fogwe and Mesfin, 2018).

In the mature brain, two types of vessels are evident: (1) *pial vessels*, either free in the subarachnoid space surrounded by cerebrospinal fluid or on the surface of the brain contacting the pia-arachnoid and the glia-limitans; and (2) *intraparenchymal vessels*. Pial vessels (intracranial vessels) are predominantly: arteries, arterioles, veins, venules and a few capillaries (Holash et al., 1990). Intraparenchymal vessels are rich in capillaries with a low permeability rate and express γ-glutamyl transpeptidase, alkaline phosphatase and enzymes involved in catecholamine metabolism. While the vascular volume of the parenchymal vessels in the cerebrum and cerebellum remains almost identical, the volume of the vasculature corresponding to the pia is 10–60% higher in the cerebellum, where pial vessels are <6% of the total cerebral vasculature and >30% of the total cerebellar vasculature (Holash et al., 1990). The blood brain barrier (BBB) formed by dynamic endothelial cells that continuously develop tight junctions to limiting cross of molecules, is absent in the vasculature near the ventricular system and in the choroid plexus, where fenestration allows exchange of nutrients (Tata et al., 2015). It is not clear if BBB possesses specific properties to maintain the homeostasis of local neural circuits; however, vascularized areas next to the 4V present widespread passive permeability (Daneman and Prat, 2015).

**Figure 2:**
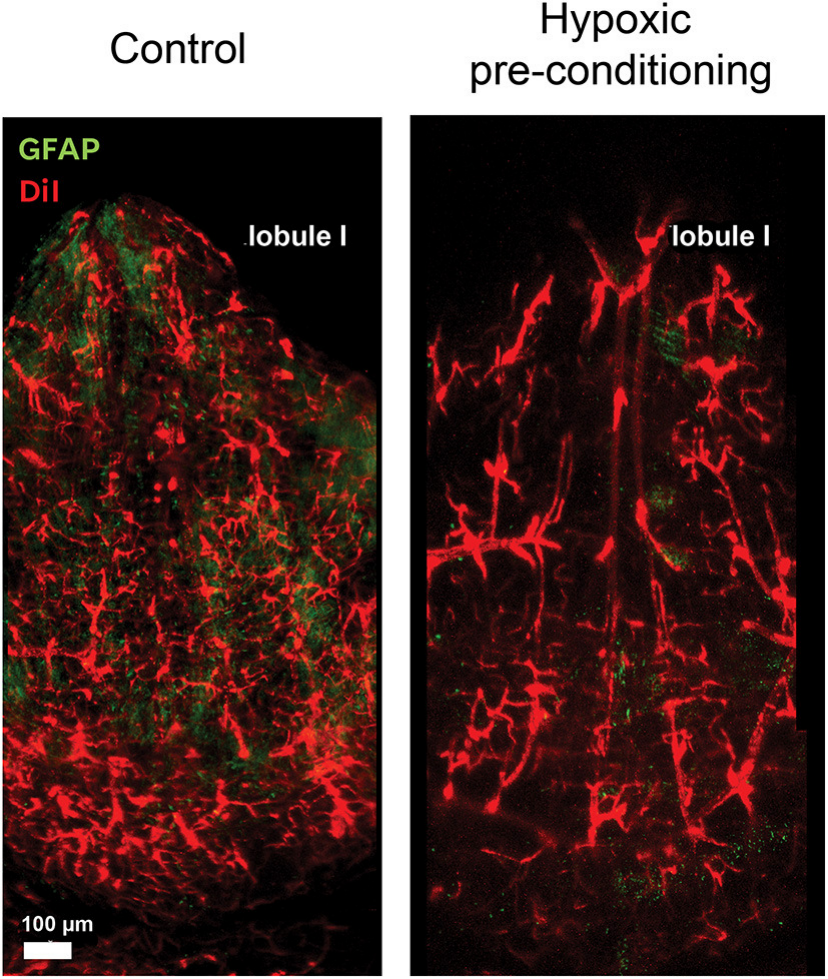
Blood vessels respond to hypoxic preconditioning. The surface of the roof of the 4V is profusely irrigated by blood vessels and arteries. These confocal microscope images show the vessels stained with DiI and the surface of the lobule I exposed by the whole mount preparation, before and four days after hypoxic pre-conditioning, where vessel dilation can be observed.

In the adult life, neuropathologies such as ischemia as well as mild to moderate reduction of oxygen tension (hypoxic pre-conditioning) promote angiogenesis. Moreover, hypoxic pre-conditioning enhances angiogenesis and confers neuroprotective effects due to the generation of new blood vessels which in turn provide oxygen and nutrients to the brain (Li et al., 2013; Hoffmann et al., 2015). Studies in our laboratory demonstrated that hypoxic pre-conditioning induces the widening of the vasculature on the surface of the roof of the 4V. This response may be associated to the metabolic modifications in the environment that led to the reconfiguration of the local glial morphology, which includes BG, astrocytes and microglia. Images of the blood vessels from the 4V, before and after hypoxic preconditioning are shown in Figure 2. In this experiment GFAP-eGFP mice (Nolte et al., 2001) were perfused with the fluorescent lipophilic membrane dye DiI for labeling the vasculature and then fixed with paraformaldehyde. The cerebellum was removed, and the ventricular surface exposed for evidencing the vasculature. After 4 days of hypoxic conditioning, the vessels were considerable dilated; this was an expected effect related to mild hypoxia, in which robust vascular remodeling occurs.

## The neural component

We are usually familiarized with the concept that the cerebellum has a uniform set of layers with homogeneous cytoarchitectural organization along the lobules. This is mostly true, but peculiarities emerge when carefully analyzing the surface in contact with the 4V. Each zone of the cerebellar periventricular area has a specific neuronal population and therefore functions that contribute to the CNS homeostasis.

### Subventricular zone

Axons from the descending section of the fastigial nucleus (FN) cross contralaterally along the roof of the ventricle mainly projecting to the vestibular system (Walberg et al., 1962; Voogd, 1967; Voogd and Glickstein, 1998; Gómez-González and Martínez-Torres, 2021). These axons are generally formed by neurons with a dense myelin sheath, and show diverse neurochemical identity either glutamatergic, glycinergic or GABAergic (Robinson et al., 1993; Uusisaari et al., 2007; Bagnall et al., 2009). Interestingly, in a study conducted by Gómez-González and Martínez-Torres (2021), it was demonstrated that the medial portion of the FN located between the cerebellar lobules I-II, form an inter-fastigial direct pathway composed mostly by GABAergic axons, adding complexity to this circuit. Functionally, the rostral region of the FN is associated with regulation of ocular movements (initiation, consistence, and accuracy of saccades), in addition its stimulation increases the systemic blood pressure (Nisimaru and Kawaguchi, 1984; Robinson et al., 1993; Takahashi et al., 2014).

The dorsal portion FN gives rise to the *locus coeruleus*, that is formed by A4 neurons (Grzanna and Mottiver, 1980), which are localized in the lateral region of the subventricular zone of the 4V. These dopamine β hydroxylase positive neurons, with spindle-shaped soma (Grzanna and Mottiver, 1980), run longitudinally and extend their dendrites through the ependymal cells toward the ventricular lumen (Demirjian et al., 1976).

### Periventricular zone

A neuronal network called the cerebrospinal fluid-contacting neurons (CFCN) reaches the lumen of the ventricle with their axon or dendrites, forming neurohormonal nerve terminals and sensory cilia (Vigh-Teichmann and Vigh, 1983; Vígh et al., 2004); the long single-cilia has a 9 × 2+2 structure. These neurons are GABA^+^ in small mammals (mouse and rats), and AChE^+^ in fish (Vigh-Teichmann and Vigh, 1983). The function of these neurons has not been elucidated, nevertheless are associated with the sensing of CSF pressure, flow and composition (Vígh et al., 2004), maintaining the homeostasis of the whole brain by non-synaptic transmission, since the neurohormonal terminals release bioactive substances to the CSF.

### Supraventricular zone

This zone includes the *vellum medullaris* and choroid plexus. The *vellum medullaris* consists of a sheet of tissue attached to the inferior colliculus and the cerebral vermis (Louvi et al., 2003). The anterior section of the vellum is located at the anterior section of the vermis and continues to the roof throughout the rostral region of the 4V, linking both cerebellar peduncles (Berry et al., 1995). It is a reminiscent zone that derives from the isthmus during embryonic development (Louvi et al., 2003). The anterior section of the vellum presents myelinated and non-myelinated axons (Berry et al., 1995) that arise from the fourth cranial nerve nucleus and project contralaterally to the IVth nerve rootlets rising to the trochlear nerve (Sliney et al., 1981; McConnell et al., 1984; Berry et al., 1995). Finally, this nerve enters to the ocular orbit by passing through the superior orbital fissure to innervate the superior oblique muscle (Brazis, 2014).

## Conclusions

**Figure 3:**
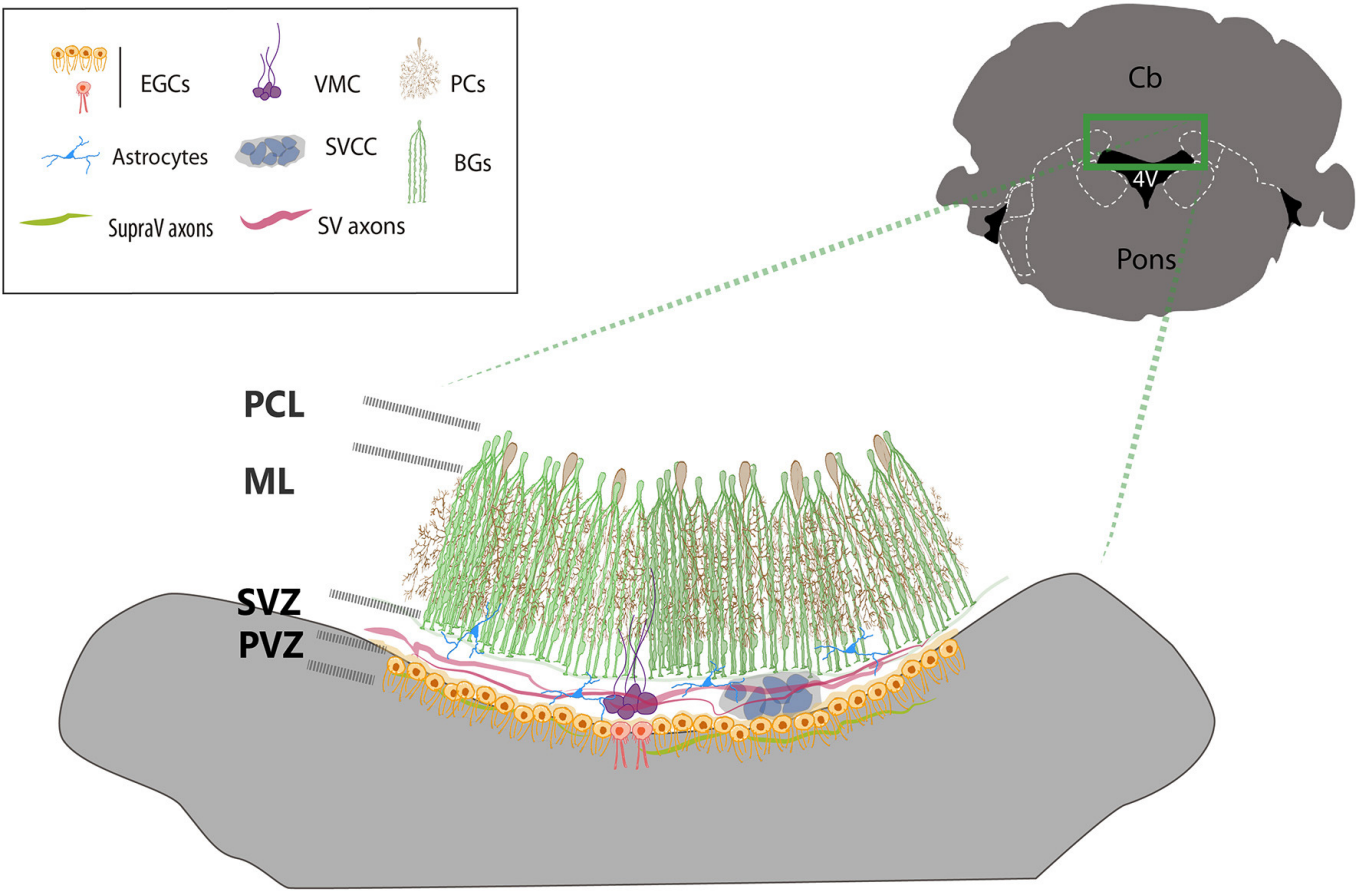
Cellular organization of the ventricular zone of the cerebellum. The drawing summarizes the main cell types found in the roof of the 4V. BG cells (green) surrounds and covers the dendritic trees of Purkinje neurons (brown). The end-feet of Bergmann glia borders the dorsal side of the SVZ, where the subventricular cellular cluster is located (SVCC, gray blue) which is ventrally bordered by multiciliated ependymal glial cells (orange cells). The ventromedial cord (VMC, purple), is integrated by local subventricular neurons and glial cells and contacted by local astrocytes (blue). Ependymal biciliated cells (red) are in the midline at the periventricular layer-zone (PVZ), facing the lumen of the ventricle. Two sets of axons transverse contralaterally the roof of the 4V, subventricular axons (SV axons, red) from the fastigial nucleus and supraventricular axons (SupraV axons, green) form the *vellum medularis* that project from the IV cranial nerve.

This brief analysis of the organization and function of the cells that form the roof of the 4V suggests that the area drastically differs from the rest of the lobules of the cerebellum. The microenvironment generated by the CSF, EGCs, choroid plexus, and blood vessels put forward an argument in favor of the peculiarities of the zone (summarized in Figure 3). There are major discrepancies yet to be resolved; for example, the diversification of functions of the EGCs regarding to their responses to GABA, cilia organization and distribution, the functional organization of axons crossing the roof of the 4V, the role of local non-myelinated neurons, the functional significance of the morphological varieties of glia and microglia among other stimulating questions worth to be explored with new experimental strategies.

## Author contributions

AM-T conceived and designed the entire review. All authors wrote, reviewed, edited, and read and approved the manuscript.

## Funding

This review was supported by Grants from CONACYT (A1S7659) and PAPIIT-DGAPA IN204520 to AM-T.

## Conflict of interest

The authors declare that the research was conducted in the absence of any commercial or financial relationships that could be construed as a potential conflict of interest.

## Publisher's note

All claims expressed in this article are solely those of the authors and do not necessarily represent those of their affiliated organizations, or those of the publisher, the editors and the reviewers. Any product that may be evaluated in this article, or claim that may be made by its manufacturer, is not guaranteed or endorsed by the publisher.
